# A risk assessment of equine piroplasmosis entry, exposure and consequences in the UK

**DOI:** 10.1111/evj.13579

**Published:** 2022-05-22

**Authors:** Robert M. Coultous, David G. M. Sutton, Lisa A. Boden

**Affiliations:** ^1^ Institute of Biodiversity Animal Health and Comparative Medicine, College of Medical, Veterinary and Life Sciences University of Glasgow Glasgow UK; ^2^ School of Veterinary Medicine, College of Medical, Veterinary and Life Sciences University of Glasgow Glasgow UK; ^3^ Global Academy of Agriculture and Food Security The Royal (Dick) School of Veterinary Studies and The Roslin Institute Midlothian UK

**Keywords:** *Babesia caballi*, horse, piroplasmosis, risk assessment, *Theileria equi*, UK

## Abstract

**Background:**

Equine piroplasmosis (EP) is currently not endemic in the UK, despite a lack of formal surveillance and the presence of carrier horses in the equine population. Pathogen establishment would have significant welfare and economic impacts on the national equine industry, but the disease is often overlooked by UK practitioners.

**Objectives:**

To assess the risk of disease entry, exposure and consequences to the UK equine population.

**Study design:**

Qualitative risk assessment.

**Methods:**

A qualitative risk assessment was constructed utilising the current World Organisation for Animal Health (OIE) published framework for importation risk assessment, assessing the key areas of disease entry, exposure and consequences to the UK equine population.

**Results:**

The overall risk of EP entry to the UK via importation of infected equidae with acute disease is very low but considered medium with subclinical carrier animals. Entry via importation of ticks or the importation of blood is considered very low. The risk of EP exposure to susceptible equidae in the UK is considered low by the infection routes of tick‐bites, contaminated needles and contaminated blood, but very high via transplacental transfer. However, the consequences of EP endemic establishment are considered of high significance to the UK equine industry.

**Main limitations:**

A lack of available numerical data for events and variables in disease import risk meant a qualitative assessment was the most practical method for this scenario.

**Conclusions:**

This risk assessment highlights that EP positive animals are able to enter and are currently present in the UK, and that conditions do exist that could allow forward transmission of the disease. It has highlighted a gap in existing policy where the UK falls behind OIE guidelines and has suggested steps to correct this discrepancy and improve national biosecurity.

## 1 INTRODUCTION

Equine piroplasmosis (EP) results from single or co‐infection with the intracellular blood apicomplexan parasites *Theileria equi* and *Babesia caballi* and is a disease of global welfare and economic significance.^1^ Only a tenth of the global equine population is estimated to live in areas free of endemic disease,^2^ including the UK, Ireland, USA, Australia and Japan.

EP is transmitted through the bite of infected tick vectors, however artificial transmission through iatrogenic blood‐to‐blood contact is possible. Clinical disease is similar regardless of the infecting parasite species and presents in both acute and chronic forms. Acute cases typically present with pyrexia, lethargy, dehydration, anorexia and anaemia, with death occurring in severe or neglected cases.^3^ In the chronic form, clinical signs are milder with animals displaying fluctuating malaise, anorexia, weight loss or reduced performance.^2^, ^4^ Infected animals become carriers, acting as reservoirs for further infection. This carrier status can persist for years^5^ or life,^6^ even continuing despite medical treatment,^7^ with potential for acute recrudescence in times of co‐infection or stress.

Equine piroplasmosis has serious effects on the reproductive capacity of an equine population. Transplacental transmission of *T. equi* in infected mares can result in abortion, fatal neonatal piroplasmosis at birth or the offspring being born a sub‐clinical carrier of disease.^8^, ^9^, ^10^ Ultimately the disease has significant implications for animal welfare and fertility as well as local and global equine industry and trade.

Historically, EP has not established in the UK due to a lack of competent tick vector species.^11^ However, *Dermacentor reticulatus*, a major EP vector species, is increasingly being identified as endemic in some areas of the British Isles.^12^

The UK does see positive cases associated with imported animals,^9^, ^13^ but tick‐borne transmission within the British Isles has yet to be recorded despite the presence of EP positive animals.

Unlike other EP‐free countries, there is no official obligation to report the disease in the UK, and UK cases are only monitored indirectly via EP diagnostic submissions to laboratories contributing to the Equine Quarterly Disease Surveillance Report.^14^

An import risk analysis encompasses the steps of hazard identification (the pathogen or risk to be considered), risk assessment (entry, exposure and consequence assessment and risk estimation associated with the hazard), risk management (options available to manage the hazard) and risk communication (information exchange with affected parties and stakeholders).^15^

Here the authors focus primarily on the risk assessment component of the analysis, with the purpose of highlighting the most important risks and consequences to be considered by veterinarians and individuals involved in equine importation to the UK. Consequently, the goals of this qualitative risk assessment (QRA) are to estimate the likelihood and consequences of introduction and onward spread of EP into the UK and discuss recommendations to mitigate the threat.

## 2 MATERIALS AND METHODS

This QRA addressed the specific risk question ‘What is the current risk of EP being introduced to the UK and disease becoming endemic in UK resident equines?’ providing a detailed account of entry and exposure pathways, and an assessment of consequences from EP introduction.

Qualitative risk assessments express the likelihood of a risk using words (e.g. negligible to high risk), in contrast to quantitative risk assessments which uses numerical measures, such as the number and frequency of events, to numerically express the probability of a risk and the magnitude of its effect.

At present, there is lack of either available, comprehensive or up‐to‐date numerical data for UK equine and tick population size and distribution, the current prevalence of EP in UK resident equines, the efficacy of EP transmission by indigenous UK tick species, and many other important variables surrounding UK EP entry and transmission. Consequently, this disease import risk assessment necessitated a qualitative rather than quantitative risk assessment approach for the UK situation.^16^

This QRA was constructed utilising the comprehensive World Organisation for Animal Health (OIE) published framework and guidance for importation risk assessment^15^ which is adopted by the UK Department of Environment, Food and Rural Affairs (DEFRA).^17^

This QRA follows three key areas of the OIE framework:

- *Entry assessment* ‐ What is the probability of EP entering the UK from EP disease endemic countries?
- *Exposure assessment* ‐ What is the probability of EP transmission to susceptible animals in the UK?
- *Consequence assessment* ‐ What are the consequences of EP introduction to the UK?

**FIGURE 1:**
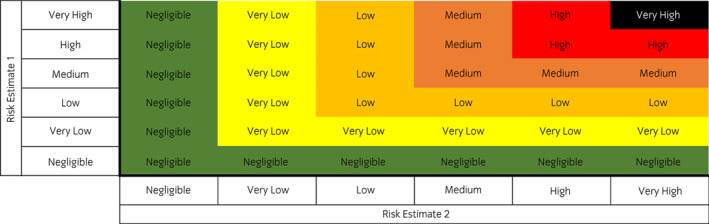
Evaluation matrix for the product of two risk estimate probabilities^16^

Each element of this QRA is provided with an assessed level of qualitative likelihood using accepted terminology definitions derived from those published by the OIE^15^ and adopted by DEFRA,^17^ in addition to an indicated level of uncertainty for the given level, based on previously established definitions^18^ (Table 1). Where applicable, overall risk estimates probabilities were combined using a previously described matrix^16^ (Figure 1).

**TABLE 1:** Terminology for assessed level of risk and uncertainty level^16^, ^18^

Risk term	Definition
Negligible	So rare it does not merit to be considered
Very Low	Very rare but cannot be excluded
Low	Rare but does occur
Medium	Occurs regularly
High	Occurs often
Very high	Event occurs almost certainly

Uncertainty category	Interpretation
Low	There are solid and complete data available; strong evidence is provided in multiple references; authors report similar conclusions
Medium	There are some but no complete data available; evidence is provided in small number of references; authors report conclusions that vary from one another
High	There are scarce or no data available; evidence is not provided in references but rather in unpublished reports or based on observations, or personal communication; authors report conclusions that vary considerably between them

## 3 RESULTS

### 3.1 Entry assessment

**FIGURE 2:**
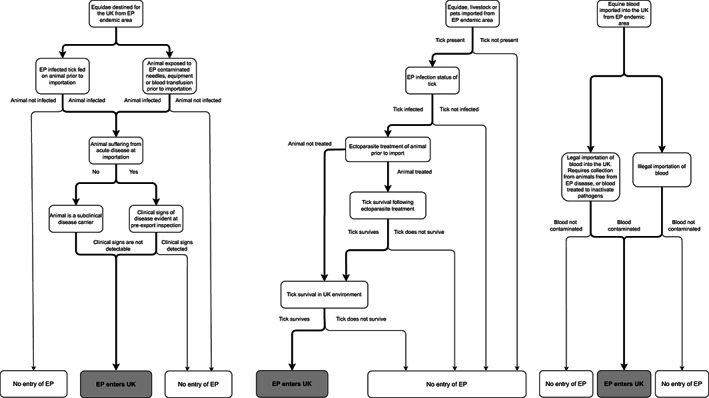
Risk pathways for EP entry

Three likely risk pathways for EP entry into the UK have been identified (Figure 2). A summary of entry pathway risks, described in detail below, is presented in Table 2.

**TABLE 2:** Summary of EP entry pathway risks

Entry pathway	Entry circumstance	Possibility of event	Occurrence likelihood		Overall entry risk estimate	Level of uncertainty for risk estimate
Infected equidae	Acute disease	**Very low**	Likelihood of equidae imported from endemic areas being EP positive	**Medium**	**Very low** (Acute disease)	Medium
	Subclinical disease	**Very high**			**Medium** (Subclinical disease)	
Infected tick	Imported on equidae	**Low**	Tick survival if *D. reticulatus*	**Low**	**Very Low**	Medium
	Imported on cattle	**Very low**				
	Imported on other ungulate	**Low**	Tick survival if other species	**Very low**		
	Imported on pet	**Low**				
Infected blood	Legal importation of infected whole blood	**Very low**	Blood importation is highly restricted	**Very low**	**Very Low**	Low
	Illegal importation of infected whole blood	**Medium**	Illegal blood importation is infrequent	**Low**		

*Note*: Risk estimates are highlighted in bold.

#### 3.1.1 Importation of EP infected carrier equidae

##### What is the likelihood of an EP infected animal being able to enter the UK?

There are currently no formal requirements to screen equidae for EP prior to or at importation. However, animals are required to be certified free of clinical disease following clinical examination at a pre‐export inspection by an official veterinarian of the exporting country on the day of loading for export^19^ and declared by the owner or breeder to have been free from contact with equidae suffering from contagious or infectious disease in the 15 days prior to examination.^20^ Additionally, animals may be subject to post‐import inspection at UK Border Control Posts.^19^ It is likely that these veterinary inspections and declarations would be effective in detecting animals suffering from acute EP disease given the severity of clinical signs observed in these cases, but carrier animals with sub‐clinical disease would not be detectable by these means.

A recent survey of UK equine samples submitted for EP screening reported a seroprevalence of 8%, with EP parasite DNA detectable by PCR in 0.8% of samples,^21^ demonstrating that EP carrier animals are present in the UK. EP has been ranked the sixth most frequent disease incident resulting from international horse movement^22^ and the OIE ‘High Health, High Performance’ (HHP) scheme, which facilitates the movement of competition horses for international events whilst maintaining equine health biosecurity, considers EP to be a transmission risk even when the compartmentalisation and biosecurity principles of the scheme are observed.^23^ Therefore, while the likelihood of an animal with acute EP disease entering the UK is very low, the likelihood of a sub‐clinical EP carrier animal being able to enter is very high.

##### What is the likelihood of an animal imported to the UK from an endemic area being EP positive?

The likelihood of an imported individual being EP positive will vary with the country of origin and previous travel history, in Europe alone overall seroprevalence of *T. equi* and *B. caballi* in endemic countries is 30% and 8% respectively, and PCR prevalence is 25% and 2% respectively.^24^ Due to the high financial costs involved in international equine transport, it is assumed that frequently travelled performance animals and high value animals represent the greatest proportion of imported equines, and these animals are often subject to more stringent biosecurity controls, such as the HHP scheme,^23^ to reduce the risk of disease dissemination. However, a lack of published data gives this assumption a high uncertainty level.

It is concluded that the risk of an imported equid being EP positive is medium, although may vary from low to high depending on the animal's travel history.

The overall risk estimate of EP into the UK is considered very low in case of animals with acute disease, but medium through the introduction of animals with subclinical disease.

#### 3.1.2 Importation of EP infected ticks

##### What is the likelihood of an EP infected tick being able to enter the UK?

There are five known genera of ticks that can act as EP vectors; *Amblyomma* spp., *Dermacentor* spp., *Haemaphysalis* spp., *Hyalomma* spp. and *Rhipicephalus* spp.^25^ All are multi‐host ticks and in EP endemic areas feed on cohabiting non‐equine animals, and these animals may also act as a vehicle to transport an EP positive tick into the UK.^26^, ^27^

Ectoparasiticide treatment of equidae is not required before UK importation.^19^ The probability of detecting ticks on an EP carrier animal during the pre‐export inspection would depend on the anatomical location, number and age of the ticks present. Instances have been documented of adult ticks from EP competent species being detected on horses after UK importation.^28^ Therefore, there is a low risk of an EP infected tick entering the UK on imported equidae.

All bovine animals imported to the UK are also subjected to a compulsory pre‐export veterinary examination, with additional compulsory ivermectin treatment for the prevention of warble fly immediately post‐import.^29^ Although ivermectin is not directly licensed for use against ticks, there is evidence of activity against a range of tick species.^30^ Consequently, the risk of these species transporting an EP infected tick into the UK would be very low.

Ovine, caprine and other ungulate imports are also subject to the same pre‐export inspection, but not compulsory warble fly treatment.^31^, ^32^ There is therefore a low risk of an EP infected tick being imported with these animals.

Pet canines and felines require a pet passport or animal health certificate prior to UK entry^33^ but compulsory tick treatment of pets has not been required since 2012.^34^ Four of the tick genera capable of EP transmission have been identified on companion animals travelling to the UK,^35^ generating a low risk that these animals may act as a vehicle for the importation of an EP infected tick.

##### What is the likelihood of an EP infected tick surviving in the local ecosystem following importation to the UK?

All the known EP tick vector genera have been detected on animals entering the UK. Apart from *Dermacentor reticulatus*, which has limited UK distribution in west Wales, Devon and Essex,^12^ there is no evidence for the on‐going survival and establishment of EP tick vectors in the UK.^36^

Consequently, the likelihood of *D. reticulatus* surviving would be low, although this would vary with climate, season and geography,^37^ with survival in southern rural parts of the UK more likely. The risk of other tick vector species surviving would be considered very low.

In summary, there is a very low risk of EP entering the UK via an infected tick.

#### 3.1.3 Importation of EP infected equine blood

Transfusion with EP infected equine blood is a viable method of transmission.^7^, ^38^ The importation to the UK of whole equine blood is tightly regulated due to concerns over African Horse Sickness and other pathogens. Importation of equine blood products for purposes other than research require specific authorisation from the Animal and Plant Health Agency (APHA) Import of products, animals, food and feed system (IPAFFS).^39^

General licences for research purposes are available for importation of heat treated blood,^40^ but certain samples may require specific APHA authorisation.^41^

The stringent controls on importation of blood from equidae mean the risk of importing EP via infected blood is very low. However illegal importation of blood poses an ever present disease risk, as demonstrated with the 2006 EIA outbreak in Ireland resulting from the illegal importation of equine serum from Italy.^42^ The risk of illegally imported blood being EP infected is considered medium.

The overall risk of EP entry via infected blood should be considered very low.

### 3.2 Exposure assessment

**FIGURE 3:**
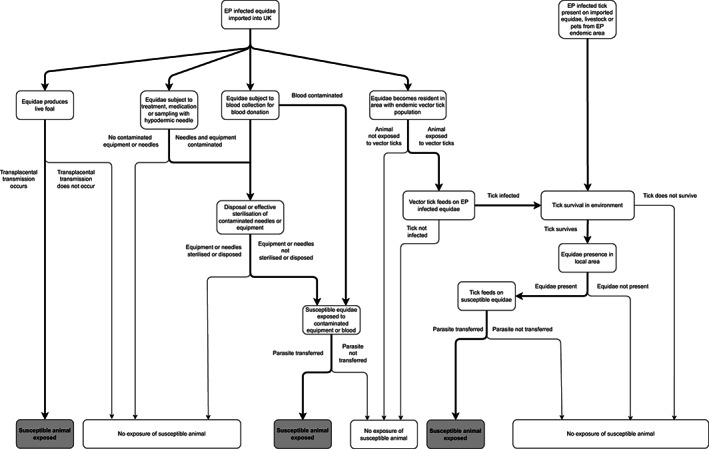
Risk pathways for EP exposure

Following entry of EP by either an equine or tick carrier, exposure and infection of susceptible equidae in the UK may occur by four possible routes (Figure 3). A summary of the risk of exposure and infection through each exposure pathway is shown in Table 3.

**TABLE 3:** Summary of exposure risk. Note that the exposure and infection risk are not multiplicative, i.e., a pathway may have a low exposure risk if the exposure by that pathway is unlikely, however, if exposure by that pathway does occur, the likelihood of infection could be high

Exposure pathway		Risk of exposure	Uncertainty of risk estimate		Risk of infection/contamination	Uncertainty of risk estimate
Exposure of vectors and equipment to infection or contamination from EP infected animals						
Infection of native ticks	*D. reticulatus*	**Low**	Medium		**Medium**	Medium
	Other UK tick spp.	**Negligible**	Medium		**Negligible**	Low
Contamination of equipment	Hypodermic needles and syringes	**Medium**	Low		**High**	Low
	Other equipment	**Medium**	Low		**High**	Medium
Exposure of susceptible equidae						
Equidae exposure via tick		**Low**	Medium		**Medium**	Low
Equidae exposure via contaminated equipment	Hypodermic needles and syringes	**Low**	Medium		**High**	Low
	Other equipment	**Low**	Medium		**Very low**	Medium
Equidae exposure via contaminated blood		**Low**	Medium		**Very high**	Low
Equidae exposure via transplacental transmission		**Very high**	Low		**High**	Low

*Note*: Risk estimates are highlighted in bold.

#### 3.2.1 Vector‐borne transmission

##### What is the likelihood of UK ticks acquiring EP infection from an imported carrier animal?

*Dermacentor reticulatus* is the only indigenous UK tick species known to be capable of EP transmission and has a limited distribution.^12^ An imported carrier animal would have to become resident in an area with an active population of ticks to increase the risk beyond a negligible level.

Transmission of EP from a carrier animal to *D. reticulatus* through tick feeding has not been directly studied, but studies of other tick species have shown efficiencies of between 7%–50%.^43^ Although the efficiency of transmission to *D. reticulatus* is highly uncertain, given the high rate of parasite acquisition in other tick species, the risk of a carrier animal transmitting EP when in contact with *D. reticulatus* should be considered medium. However, the overall risk of UK *D. reticulatus* populations acquiring EP infection from a carrier animal should be considered low. The risk of other indigenous tick species acquiring EP is negligible due to their presumed lack of vector capacity.

##### What is the likelihood of an EP infected tick transmitting infection to susceptible equidae?

At present there has been no reported tick transmission of EP within the UK. Established populations of *D. reticulatus* are restricted to west Wales, Devon and Essex,^12^ with the latter linked to an outbreak of canine piroplasmosis.^44^ The UK currently operates the Public Health England's Tick Surveillance Scheme (TSS).^45^ This passive scheme relies on voluntary submissions from members of the public, veterinarians, health and wildlife workers.^46^ The limitations of the TSS mean other undocumented populations of *D. reticulatus* may exist in cohabitation with equine populations.

There are some known equine populations that geographically overlap the limited distribution of *D. reticulatus*, namely the semi‐feral grazing equine populations of Exmoor and Dartmoor. These horses constitute significant populations; over 500 registered animals on Exmoor, although this is widely accepted to be a substantial underestimate due to the problem of horse abandonment on the moor,^47^ and an estimated 1500 animals on Dartmoor.^48^ With no formal surveillance of either population numbers or EP disease within these animals, they form an area of potential EP exposure and disease reservoir.

The life cycle of *D. reticulatus* also has an influence on exposure risk. The tick is extremely hardy, able to live up to 4 years without a blood meal, remaining active during the winter and able to survive prolonged periods below −0°C.^37^ Consequently, EP positive ticks could provide an environmental threat to grazing animals for a number of years.

There are no specific data regarding *D. reticulatus*, but other *Dermacentor* species can transfer *B. caballi* from one generation of tick to the next via transovarian transmission,^49^ and depending on the tick species, this can continue for several generations without an equine host.^50^, ^51^ With *D. reticulatus* females producing between 2200–7200 eggs at a time,^52^ there is the potential for amplification of *B. caballi* in the environment. Note that tick transovarian transmission does not occur with *T. equi*.^53^

The susceptibility of naïve equidae to tick‐borne EP is well documented^38^ and a 2009 outbreak in Texas, USA, saw the efficacy of tick driven EP spread result in 81.1% EP seropositivity within exposed horses over a period of months.^54^ In that locality a novel vector *Amblyomma cajennense* was identified as the primary tick vector, highlighting that endemic tick populations may assume increased importance after arrival of the pathogen in a new locality.

Given the limited existence of UK equid populations in areas of vector tick populations,^12^ the risk of a susceptible animal being exposed to an EP infected tick should be considered low.

However, the risk of EP infection by an infected tick feeding on a susceptible equid should be considered medium.

#### 3.2.2 Mechanical transmission

##### What is the likelihood of hypodermic needles and equipment becoming contaminated by an EP carrier animal?

Legitimate routine procedures such as diagnostic sampling, treatment and vaccination, along with other illicit practices,^55^ provide opportunity for needle and equipment contamination.

Additionally, EP carrier animals may suffer recrudescent parasitaemia with clinical signs at times of increased physical^56^ or metabolic stress,^57^ increasing the likelihood of EP carrier animals requiring veterinary interaction and providing opportunity for blood contamination of hypodermic needles and equipment.

Therefore, the general risk of hypodermic needles and equipment being exposed to EP infected blood should be considered medium, and the risk of contamination following use on known EP carrier animals should be considered high.

##### What is the likelihood of EP contaminated needles or equipment transferring infection to a susceptible equid?

Disposable syringes and hypodermic needles are both inexpensive and readily available in the UK, as are pathways for their safe disposal. It is assumed there is widespread good practice among veterinary professionals regarding needle hygiene and disposal.

However, episodes of poor practice do occur; a Newmarket equine fertility unit during the 1990s saw EP transmission to 61 of 66 animals due to syringe sharing,^13^ and the sharing of needles between horses has been implicated in disease spread in Australia^58^ and the USA.^55^, ^59^

There have been no recorded instances of disease transmission via equipment other than needles and syringes.

The risk of susceptible equidae being exposed to contaminated needles and equipment is considered low. Given previous reports, the risk of infecting a susceptible animal with a contaminated needle or syringe should be considered high. The risk of EP infection via other blood‐contaminated equipment should be considered very low.

#### 3.2.3 Haematological transmission

##### What is the likelihood of a susceptible equid being infected through transfusion of EP infected blood?

The intravenous transfusion of naïve animals with infected blood is highly efficient and the preferred method of inoculation for experimental infection studies.^7^, ^60^ Blood and plasma transfusions are performed in general equine veterinary practice and in equine hospital facilities. Due to the potential for transfer of a wide range of diseases, good practice requires the use of blood donors with known medical history and no history of foreign travel.^61^

The illegitimate practice of blood doping was linked to a 2008 EP outbreak in Florida resulting in EP transmission from two illegally imported Mexican horses spreading to 20 horses across 7 premises.^55^ The illicit nature of this practice makes quantifying the significance of this risk difficult, although it is assumed that such practices are not widespread in the UK.

There has been no study of the capability of EP to survive storage in blood bags, but another piroplasm, *Babesia microti*, can remain infective in blood stored at 4°C for up to 21 days.^62^ It is assumed that this may apply to *T. equi* and *B. caballi*, but the uncertainty of this statement is high.

Consequently, the risk of susceptible equines being exposed to EP infected blood should be considered low, however the risk of susceptible equidae becoming infected from the transfusion of EP infected blood should be considered very high.

#### 3.2.4 Transplacental transmission

##### What is the likelihood of an unborn foal being born infected from an EP carrier dam?

*Theileria equi* can be transmitted transplacentally to foals from as early as month four of gestation^10^ and cause fatal haemolytic disease at birth. *Theileria equi* carrier mares have also been shown to birth foals that are born healthy but are EP carriers, with transplacental transfer of infection demonstrated in all clinically healthy foals from six carrier mares in one study.^10^ Carrier mares can continue to birth EP positive foals for several years.^9^

All foals born to an EP infected dam are exposed, so exposure risk should be considered very high. Given an apparently high rate of infection, the risk of neonate infection from an infected dam is high.

#### 3.2.5 Consequence assessment

**FIGURE 4:**
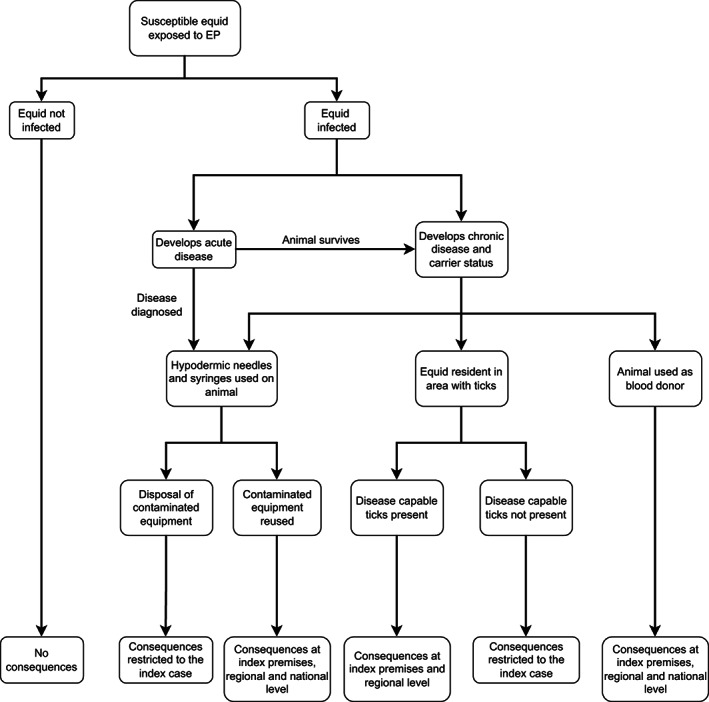
Consequences of EP introduction through identified risk pathways for exposure

An outbreak via the described exposure pathways carries direct and indirect consequences (Figure 4).

#### 3.2.6 Direct consequences

##### Outcome of exposure in animals

The risk of an animal being infected in the UK is low. However, the severity of the consequences will vary between individuals.

Considering acute disease cases, outbreaks in Florida 2008^55^ and Texas 2009^54^ show the number of animals presenting with clinical signs of acute disease is likely to be very low; only 3% of exposed horses in Florida and 0.3% of exposed horses in Texas demonstrated acute disease. However the consequences for animals with acute disease are likely to be severe, with mortality rates as high as 69% observed in untreated naïve animals.^63^

Mortality within those animals developing chronic disease or carrier status is likely to be very low, only occurring in the event of acute disease recrudescence.

However, the insidious nature of EP transmission means a significant morbidity rate is likely by the time of outbreak investigation; 28 of 60 exposed horses in the Irish 2009 County Meath outbreak were seropositive by the time of investigation.^64^ Mechanical transmission in the 2008 Florida outbreak saw 20 of 210 exposed horses seropositive by the time of disease detection,^55^ and in the 2009 Texan outbreak, caused by tick transmission, resulted in 292 of 360 horses being seropositive by the time the first clinical case was detected and investigated.^54^

Carrier horses are most significant in terms of endemic EP disease risk, especially since lifelong infection with *T. equi* provides a persistent reservoir of disease.^6^

##### Production and performance losses

Despite low mortality in chronic cases, productivity losses can be significant. In South Africa, EP is a common cause of abortion^2^ with up to 11% of abortions in Thoroughbred mares attributed to *T. equi*.^8^

There is also concern regarding reduced athletic performance in carrier animals,^10^, ^56^ with South African reports suggesting around 2% of young stock are lost from the industry before their first race due to EP related poor performance or disease,^65^ although the effect of EP status on performance has recently been debated.^66^

Given the potential for a high rate of morbidity before disease is detected, chronic EP infection poses the most likely threat of high direct consequences on both productivity and performance.

#### 3.2.7 Indirect consequences

##### Effects on domestic equine industry and trade

The UK has a significant equine industry^67^ and establishment of endemic EP would extend beyond the welfare and economic costs of clinical disease and lost production. Additional costs of routine diagnostics for disease surveillance would be compounded with those of preventative measures such as tick control, increasing financial overheads for the equine industry in the form of ectoparasiticide use and changes in land management.

Thus, the economic consequences of endemic EP to the UK equine industry would be high.

##### Effects on international equine trade

A UK EP outbreak may lead to countries banning equine imports, such as seen with a 5‐month Japanese ban of French imports in February 2016 following the identification of EP‐seropositive foals in a French shipment.^68^

Establishment of EP within the UK equine population, and subsequent change in national disease status, would not only restrict the number of animals available for export to certain countries, but potentially threaten existing movement policies.

The consequences to international trade following an EP outbreak should be considered high to very high depending on the extent of the incident.

##### Disease control and eradication costs

Florida provides a pertinent example of tick‐born EP eradication difficulties and expense. Following EP introduction with imported Cuban horses, an intense state eradication programme was initiated in 1962, with eradication only declared 26 years later at a total cost of over $12 million to the state.^69^ Rapid containment and eradication of EP incursion has only ever been achieved when spread has occurred via mechanical methods, such as the later 2008 Florida outbreak.^55^

Consequently, attempts to control and eradicate an incursion of EP into the UK would have high financial and resource costs.

##### Surveillance of imported equidae

There is currently no EP screening required for entry of equidae into the UK, unlike other EP‐free countries. The USA has particularly stringent entry requirements that includes pre‐import inspection for ectoparasites and a quarantine period.^70^ Introduction of these measures to the UK would increase the cost borne by the importing parties and increase administration costs to the state. Consequently, the consequences to both the state and the UK equine industry of instigating such measures would range from medium to high depending on the complexity of the surveillance methods implemented.

##### Surveillance of EP within the UK resident equine population

Active surveillance, through the sampling and testing for EP within a population, usually only occurs during a disease outbreak.^55^ Passive surveillance usually takes the form of classifying the disease as notifiable, as in the USA^71^ and Ireland,^72^ with diagnosis or suspicion of the disease reported to the state veterinary department, where a containment plan is drawn up relative to the situation.

Making the disease notifiable in the UK, would have medium impact to the state in requiring the drafting of relevant guidelines, protocols and policy change. The implementation of active surveillance, particularly in the time of a disease outbreak, would have high or very high financial and resource consequences to the state and equine industry, dependent on nature and size of the outbreak.

##### Surveillance of ticks within the country

The TSS does not routinely test submitted ticks for pathogens, but submitted ticks are speciated and their geographical origin noted.^45^ This provides indirect monitoring for EP vector tick species. Depending on the extent of any changes to the UK's TSS, there would be medium to high financial and resource consequences to extending current tick surveillance.

## 4 DISCUSSION

This risk assessment highlights that EP positive animals are able to enter and are currently present in the UK, and that conditions do exist that could allow forward transmission of the disease. Overall, there is a low risk of disease transmission to the UK equine population, and a low risk of endemic EP establishment. However, an outbreak event may have medium to high consequences for an affected animal, and depending on circumstances, there is the potential for very high national consequences. Ultimately the consequences of unrestricted establishment of EP would be high at an individual, local and national level.

Given the assessed risk of EP disease entry, establishment and consequences in the UK, sanitary measures need to be considered to achieve a sustainable level of risk.

To comprehensively manage the risk of EP disease entry into the UK, equines need to be screened for both parasitic infection and tick infestation before being introduced to the UK equine population, as the prevention of EP entry essentially relies on the entry restriction of both EP carrier animals and EP infected ticks.

We suggest the following measures and implementation of the OIE Code recommendations^73^ to bring UK policy in line with both OIE advice and the policies of other EP‐free countries, helping to mitigate the measurable risk of EP to the UK equine population and provide improved surveillance on the number of EP carrier animals currently resident in the country and any EP outbreaks:

1. Escalation of EP to a notifiable disease status.
2. Compulsory EP serological testing of equines from an endemic country of export, performed 30 days prior to importation with a negative result required for entry.
3. Veterinary certification of equines as free from ectoparasites, preferably alongside approved acaricide treatment, in the 30 days prior to importation.

Importantly, this assessment has highlighted a gap in existing policy where the UK falls behind OIE recommendations and those of similarly positioned countries. Moving forward in the post‐Brexit environment, the UK equine industry has the possibility of reevaluating current import, export and biosecurity guidelines, giving the opportunity to review the current EP strategy to ensure the future security and welfare of its equine population.

Establishment of endemic EP in the UK would represent a significant compromise of welfare in the national equine population. Reduced fertility and athletic performance would have significant economic impacts to individual owners, breeders and trainers. The elite equine market wishing to maintain EP‐free status would face significant costs in maintaining surveillance and biosecurity within their stock and suffer severe financial and trade losses should a disease outbreak occur or, more alarmingly, should EP achieve endemic status.

## CONFLICT OF INTERESTS

No competing interests have been declared.

## ETHICAL ANIMAL RESEARCH

No ethical approval was required for this study.

## INFORMED CONSENT

Not applicable.

## AUTHORSHIP

R. Coultous contributed to conception and execution of the study, acquisition and interpretation of data, and initial drafting of the article. D. Sutton contributed to conception of the study and critical revision of the article. L. Boden contributed to the conception and design of the study and critical revision of the article. All authors have approved the submitted version of the article.

## Data Availability

Data sharing is not applicable to this article as no new data were created or analysed in this study.

## References

[1] Wise LN , Kappmeyer LS , Mealey RH , Knowles DP . Review of equine piroplasmosis. J Vet Intern Med. 2013;27:1334–46. 10.1111/jvim.12168 24033559

[2] De Waal DT . Equine piroplasmosis: A review. Brit Vet J. 1992;148:6–14. 10.1016/0007-1935(92)90061-5 1551016

[3] Wise LN , Knowles DP , Rothschild CM . Piroplasmosis. In: Sellon D , Long MT , editors. Equine infectious diseases. 2nd ed. St Louis, Missouri: Saunders Elsevier; 2014. p. 467–75.

[4] Rothschild CM . Equine Piroplasmosis. J Equine Vet Sci. 2013;33:497–508. 10.1111/eve.12070

[5] Holbrook AA . Biology of equine piroplasmosis. J Am Vet Med Assoc. 1969;155:453–4. 5816130

[6] Ueti MW , Palmer GH , Scoles GA , Kappmeyer LS , Knowles DP . Persistently infected horses are reservoirs for intrastadial tick‐borne transmission of the apicomplexan parasite Babesia equi. Infect Immun. 2008;76:3525–9. 10.1128/IAI.00251-08 18490466 PMC2493223

[7] Grause JF , Ueti MW , Nelson JT , Knowles DP , Kappmeyer LS , Bunn TO . Efficacy of imidocarb dipropionate in eliminating Theileria equi from experimentally infected horses. Vet J. 2013;196:541–6. 10.1016/j.tvjl.2012.10.025 23199699

[8] Lewis BD , Penzhorn BL , Volkmann DH . Could treatment of pregnant mares prevent abortions due to equine piroplasmosis? J S Afr Vet Assoc. 1999;70:90–1. 10.4102/jsava.v70i2.760 10855828

[9] Phipps LP , Otter A . Transplacental transmission of Theileria equi in two foals born and reared in the United Kingdom. Vet Rec. 2004;154:406–8. 15083978 10.1136/vr.154.13.406

[10] Allsopp MTEP , Lewis BD , Penzhorn BL . Molecular evidence for transplacental transmission of Theileria equi from carrier mares to their apparently healthy foals. Vet Parasitol. 2007;148:130–6. 10.1016/j.vetpar.2007.05.017 17601669

[11] Phipps LP . Equine piroplasmosis. Equine Vet Educ. 1996;8:33–6. 10.1111/j.2042-3292.1996.tb01647.x

[12] Medlock JM , Hansford KM , Vaux AGC , Cull B , Abdullah S , Pietzsch ME , et al. Distribution of the tick Dermacentor reticulatus in the United Kingdom. Med Vet Entomol. 2017;31:281–8. 10.1111/mve.12235 28419493

[13] Gerstenberg C , Allen WR , Phipps LP . The mechanical transmission of Babesia equi infection in a British herd of horses. Proceedings of the eighth international conference on equine infectious diseases. Dubai, UAE; 1998;217–22.

[14] BEVA DEFRA . Equine Quarterly Disease Surveillance Report January – March 2021;17:1. https://www.jdata.co.za/iccviewer/media/202102summ.pdf. Accessed 27 September 2021.

[15] OIE . Handbook on import risk analysis for animals and animal products: introduction and qualitative risk analysis. 2nd ed. Paris, France: World Organization for Animal Health; 2010.

[16] Kelly L , Kosmider R , Gale P , Snary EL . Qualitative import risk assessment: a proposed method for estimating the aggregated probability of entry of infection. Microb Risk Anal. 2018;9:33–7. 10.1016/j.mran.2018.03.001

[17] Auty H , Mellor D , Gunn G , Boden LA . The risk of foot and mouth disease transmission posed by public access to the countryside during an outbreak. Front Vet Sci. 2019;6:381. 10.3389/fvets.2019.00381 31750321 PMC6848457

[18] EFSA . Statement on migratory birds and their possible role in the spread of highly pathogenic avian influenza by the scientific panel on animal health and welfare (AHAW). EFSA J. 2006;4:357a. 32313578 10.2903/j.efsa.2006.357 PMC7163743

[19] APHA . Import of Equidae Import Information Note (IIN) EQ/2b, Animal and Plant Health Agency 2021 http://apha.defra.gov.uk/documents/bip/iin/eq-2b.pdf. Accessed 27 September 2021.

[20] EU . Directive 2009/156/EC on animal health conditions governing the movement and importation from third countries of equidae, European Union Council 2009. http://data.europa.eu/eli/dir/2009/156/2016-10-18. Accessed 27 September 2021.

[21] Coultous RM , Phipps P , Dalley C , Lewis J , Hammond TA , Shiels BR , et al. Equine piroplasmosis status in the UK: an assessment of laboratory diagnostic submissions and techniques. Vet Rec. 2019;184:95. 10.1136/vr.104855 30413675

[22] Dominguez M , Münstermann S , de Guindos I , Timoney P . Equine disease events resulting from international horse movements: systematic review and lessons learned. Equine Vet J. 2016;48:641–53. 10.1111/evj.12523 26509734

[23] OIE . High health, high performance (HHP) horses: risk mitigation strategies and establishment of specific health requirements. World Organization for Animal Health 2016. http://www.oie.int/fileadmin/Home/eng/Our_scientific_expertise/docs/pdf/Chevaux/HHPRiskMitigation.pdf. Accessed 27 September 2021.

[24] Nadal C , Bonnet SI , Marsot M . Eco‐epidemiology of equine piroplasmosis and its associated tick vectors in Europe: a systematic literature review and a meta‐analysis of prevalence. Transbound Emerg Dis. 2021;00:1–25. 10.1111/tbed.14261 34333863

[25] Scoles GA , Ueti MW . Vector ecology of equine piroplasmosis. Annu Rev Entomol. 2015;60:561–80. 25564746 10.1146/annurev-ento-010814-021110

[26] Fritz D . A PCR study of piroplasms in 166 dogs and 111 horses in France (march 2006 to march 2008). Parasitol Res. 2010;106:1339–42. 10.1007/s00436-010-1804-3 20221639

[27] Qablan MA , Sloboda M , Jirku M , Obornik M , Dwairi S , Amr ZS , et al. Quest for the piroplasms in camels: identification of Theileria equi and Babesia caballi in Jordanian dromedaries by PCR. Vet Parasitol. 2012;186:456–60. 10.1016/j.vetpar.2011.11.070 22186193

[28] Jameson LJ , Medlock JM . Results of HPA tick surveillance in Great Britain. Vet Rec. 2009;165:154. 10.1089/vbz.2010.0079 19648644

[29] APHA . Import of Bovine Animals Import Information Note (IIN) BVTC/2. Animal and Plant Health Agency 2021. http://apha.defra.gov.uk/documents/bip/iin/bvtc-2.pdf. Accessed 27 September 2021.

[30] Campbell WC , Benz GW . Ivermectin: a review of efficacy and safety. J Vet Pharmacol Ther. 1984;7:1–16. 6368862 10.1111/j.1365-2885.1984.tb00872.x

[31] APHA . Imports of Sheep and Goats Import Information Note (IIN) OVTC/2. Animal and Plant Health Agency 2021. http://apha.defra.gov.uk/documents/bip/iin/ovtc-2.pdf. Accessed 27 September 2021.

[32] APHA . Import of non‐domestic ungulates from third countries import information note (IIN) BLLV/3. Animal and Plant Health Agency 2021. http://apha.defra.gov.uk/documents/bip/iin/bllv3.pdf. Accessed 27 September 2021.

[33] APHA . Commercial import of dogs, cats and ferrets import information note (IIN) BLLV/5b. Animal and Plant Health Agency 2021. http://apha.defra.gov.uk/documents/bip/iin/bllv-5b.pdf. Accessed 27 September 2021.

[34] DEFRA . New rules mean it will be easier and cheaper to travel abroad with pets. Department for Environment, Food and Rural Affairs 2011. https://www.gov.uk/government/news/new-rules-mean-it-will-be-easier-and-cheaper-to-travel-abroad-with-pets. Accessed 27 September 2021.

[35] Jameson LJ , Phipps LP , Medlock JM . Surveillance for exotic ticks on companion animals in the UK. Vet Rec. 2010;166:202–3. 10.1136/vr.b4786 20154311

[36] Cull B , Pietzsch ME , Hansford KM , Gillingham EL , Medlock JM . Surveillance of British ticks: an overview of species records, host associations, and new records of Ixodes ricinus distribution. Ticks Tick Borne Dis. 2018;9:605–14. 10.1016/j.ttbdis.2018.01.011 29426591

[37] Földvári G , Siroký P , Szekeres S , Majoros G , Sprong H . Dermacentor reticulatus: a vector on the rise. Parasit Vectors. 2016;9:314. 10.1186/s13071-016-1599-x 27251148 PMC4888597

[38] Nuttall GHF , Strickland C . On the occurrence of two species of parasites in equine ‘Piroplasmosis’ or ‘biliary fever’. Parasitology. 1912;5:65–96.

[39] APHA . Import of Blood and Blood Products from Equidae for use Outside the Feed Chain from Third Countries Import Information Note (IIN) ABP/4A. Animal and Plant Health Agency 2020. http://apha.defra.gov.uk/documents/bip/iin/abp-4a.pdf. Accessed 27 September 2021.

[40] DEFRA . European Communities act 1972 ‐ the products of animal origin (third country imports) (England) regulations 2006 (as amended) general import authorisation IMP/GEN/2010/07. Department for Environment, Food and Rural Affairs 2010. https://assets.publishing.service.gov.uk/government/uploads/system/uploads/attachment_data/file/408773/IMP-GEN-2010-07-research-heat-treated-organs-glands-tissues-of-ungulate-from-certain-3rd-countries.pdf. Accessed 27 September 2021.

[41] APHA . Import of research and diagnostic samples from third countries import information note (IIN) ABP/30, Animal and Plant Health Agency 2020. http://apha.defra.gov.uk/documents/bip/iin/abp-30.pdf. Accessed 27 September 2021.

[42] More SJ , Aznar I , Bailey DC , Larkin JF , Leadon DP , Lenihan P , et al. An outbreak of equine infectious anaemia in Ireland during 2006: investigation methodology, initial source of infection, diagnosis and clinical presentation, modes of transmission and spread in the Meath cluster. Equine Vet J. 2008;40:706–8. 10.2746/042516408x363305 19165942

[43] Ueti MW , Palmer GH , Kappmeyer LS , Statdfield M , Scoles GA , Knowles DP . Ability of the vector tick Boophilus microplus to acquire and transmit Babesia equi following feeding on chronically infected horses with low‐level parasitemia. J Clin Microbiol. 2005;43:3755–9. 10.1128/JCM.43.8.3755-3759.2005 16081906 PMC1233951

[44] Sánchez‐Vizcaíno F , Wardeh M , Heayns B , Singleton DA , Tulloch JSP , McGinley L , et al. Canine babesiosis and tick activity monitored using companion animal electronic health records in the UK. Vet Rec. 2016;179:358. 10.1136/vr.103908 27484328 PMC5099196

[45] PHE . Tick awareness and the tick surveillance scheme. Public Health England 2021. https://www.gov.uk/guidance/tick-surveillance-scheme. Accessed 27 September 2021.

[46] Jameson LJ , Medlock JM . Tick surveillance in Great Britain. Vector Borne Zoonotic Dis. 2011;11:403–12. 10.1089/vbz.2010.0079 20849277

[47] Green, P. The free‐living ponies within the Exmoor National Park: their status, welfare and future. Exmoor National Park Authority 2013. https://www.exmoor-nationalpark.gov.uk/__data/assets/pdf_file/0035/286559/Final-PDF-Pony-Report-condensed.pdf. Accessed 27 September 2021.

[48] Anon . Dartmoor ponies. Visit Dartmoor Tourism Organisation 2021. https://www.visitdartmoor.co.uk/explore-dartmoor/dartmoor-animal-life/dartmoor-ponies. Accessed 27 September 2021.

[49] Neitz WO . Classification, transmission, and biology of piroplasms of domestic animals. Ann N Y Acad Sci. 1956;64:56–111. 10.1111/j.1749-6632.1956.tb36607.x

[50] Abramov IV . The duration of the preservation of the causal agent of Piroplasmosis of horses (Piroplasma caballi) in the ticks Hyalomma plumb cum panzer, 1798. J Agric Sci Moscow. 1955;32:42–6.

[51] Schwint ON , Knowles DP , Ueti MW , Kappmeyer LS , Scoles GA . Transmission of Babesia caballi by Dermacentor nitens (Acari: Ixodidae) is restricted to one generation in the absence of alimentary reinfection on a susceptible equine host. J Med Ent. 2008;45:1152–5. 10.1603/0022-2585(2008)45[1152:tobcbd]2.0.co;2 19058641

[52] Šimo L , Kocáková P , Sláviková M , Kubeš M , Hajnická V , Vančová I , et al. Dermacentor reticulatus (Acari, Ixodidae) female feeding in laboratory. Biologia. 2004;59:655–60.

[53] Mehlhorn H , Schein E . Redescription of Babesia equi Laveran, 1901 as Theileria equi Mehlhorn, Schein 1998. Parasitol Res. 1998;84:467–75. 10.1007/s004360050431 9660136

[54] Scoles GA , Hutcheson HJ , Schlater JL , Hennager SG , Pelzel AM , Knowles DP . Equine piroplasmosis associated with Amblyomma cajennense ticks, Texas, USA. Emerging Infect Dis. 2011;17:1903–5. 10.3201/eid1710.101182 PMC3310643 22000367

[55] Short MA , Clark CK , Harvey JW , Wenzlow N , Hawkins IK , Allred DR , et al. Outbreak of equine piroplasmosis in Florida. J Am Vet Med Assoc. 2012;240:588–95. 10.2460/javma.240.5.588 22332629

[56] Hailat NQ , Lafi SQ , AlDarraji AM , AlAni FK . Equine babesiosis associated with strenuous exercise: clinical and pathological studies in Jordan. Vet Parasitol. 1997;69:1–8. 10.1016/s0304-4017(96)01100-4 9187024

[57] Piantedosi D , D'Alessio N , Di Loria A , Di Prisco F , Mariani U , Neola B , et al. Seroprevalence and risk factors associated with Babesia caballi and Theileria equi infections in donkeys from southern Italy. Vet J. 2014;202:578–82. 10.1016/j.tvjl.2014.09.025 25457263

[58] Callow LL , McGregor W , Rodwell BJ , Rogers RJ , Fraser GC , Mahoney DF , et al. Evaluation of an indirect fluorescent antibody test to diagnose Babesia equi infection in horses. Aust Vet J. 1979;55:555–9. 10.1111/j.1751-0813.1979.tb07044.x 395937

[59] Knowles RC . Equine babesiasis: epidemiology, control and chemotherapy. J Equine Vet Sci. 1988;8:61–4. 10.1016/S0737-0806(88)80114-X

[60] Kuttler KL , Gipson CA , Goff WL , Johnson LW . Experimental Babesia equi infection in mature horses. Am J Vet Res. 1986;47:1668–70. 3530065

[61] Hart KA , Epstein KL . Common problems and techniques in equine critical care. In: Mair TS , Love S , Schumacher J , Smith RKW , Frazer GS , editors. Equine medicine, surgery and reproduction. 2nd ed. Edinburgh, UK: Saunders Elsevier; 2013. p. 569–71.

[62] Eberhard ML , Walker EM , Steurer FJ . Survival and infectivity of Babesia in blood maintained at 25 C and 2‐4 C. J Parasitol. 1995;81:790–2. 7472878

[63] Maurer FD . Equine piroplasmosis‐‐another emerging disease. J Am Vet Med Assoc. 1962;141:699–702. 14471543

[64] Anon. Equine piroplasmosis confirmed in Ireland. Vet Rec. 2009;165:333. 10.1136/vr.165.12.333-a

[65] Olivier A , Nurton JP , Guthrie AJ . An epizoological study of wastage in thoroughbred racehorses in Gauteng, South Africa. J S Afr Vet Assoc. 1997;68:125–9. 10.4102/jsava.v68i4.893 9561496

[66] Tirosh‐Levy S , Gottlieb Y , Steinman A . Stress conditions do not affect Theileria equi parasitemia levels in sub‐clinically infected horses. Ticks Tick Borne Dis. 2020;11:101384. 10.1016/j.ttbdis.2020.101384 32008998

[67] OIE . FAQs on high health, high performance horse (HHP) concept adopted at the may 2014 OIE general session. World Organisation for Animal Health 2014. https://inside.fei.org/system/files/FAQs_HHP_Concept_2014.docx_0.pdf. Accessed 27 September 2021.

[68] RESPE . Une cellule de crise particulière: problématique des piroplasmoses en vue d'exportation au Japon – Bulletin No 38. Réseau dEpidémioSurveillance en Pathologie Equine 2016. https://respe.net/une-cellule-de-crise-particuliere-problematique-des-piroplasmoses-en-vue-dexportation-au-japon-bulletin-n38-2. Accessed 27 September 2021.

[69] USDA . Equine Piroplasmosis and the 2010 world equestrian games. United States Department of Agriculture 2008. https://www.aphis.usda.gov/animal_health/animal_diseases/piroplasmosis/downloads/ep_2010_weg_wp.pdf. Accessed 27 September 2021.

[70] USDA . A literature review of equine Piroplasmosis. United States Department of Agriculture 2010. https://www.aphis.usda.gov/animal_health/animal_diseases/piroplasmosis/downloads/ep_literature_review_september_2010.pdf. Accessed 27 September 2021.

[71] USDA . Notifiable diseases and conditions. United States Department of Agriculture 2021. https://www.aphis.usda.gov/aphis/ourfocus/animalhealth/nvap/NVAP-Reference-Guide/Animal-Health-Emergency-Management/Notifiable-Diseases-and-Conditions. Accessed 27 September 2021.

[72] DAFM . List of Notifiable Diseases. Department of Agriculture, Food and the Marine 2020. https://www.agriculture.gov.ie/animalhealthwelfare/diseasecontrol/listofnotifiablediseases. Accessed 27 September 2021.

[73] OIE . Terrestrial Animal Health Code Chapter 12.7 Equine Piroplasmosis, The World Organisation for Animal Health 2018. http://www.oie.int/fileadmin/Home/eng/Health_standards/tahc/current/chapitre_equine_piroplasmosis.pdf. Accessed 27 September 2021.

